# Exclude or expose in irritable bowel syndrome study: what works for whom and how? Protocol for a randomised controlled trial of internet-delivered FODMAP diet versus exposure-based cognitive behavioural therapy

**DOI:** 10.1136/bmjopen-2026-126664

**Published:** 2026-11-11

**Authors:** Jacqueline L Anderson, Brjánn Ljótsson, Helen Burton-Murray, Madeline L West, Chu K Yao, Chamara Basnayake, Lukas Van Oudenhove, Jessica R Biesiekierski

**Affiliations:** 1 Human Nutrition Group, Faculty of Science, The University of Melbourne, Melbourne, Victoria, Australia; 2 Department of Clinical Neuroscience, Division of Psychology, Karolinska Institute, Stockholm, Sweden; 3 Harvard Medical School, Boston, Massachusetts, USA; 4 Center for Neurointestinal Health, Massachusetts General Hospital, Boston, Massachusetts, USA; 5 Department of Gastroenterology, Alfred Health, Monash University, Melbourne, Victoria, Australia; 6 Department of Gastroenterology, Eastern Health Clinical School, Monash University, Melbourne, Victoria, Australia; 7 Department of Medicine, The University of Melbourne, St Vincent’s Hospital, Melbourne, Victoria, Australia; 8 Laboratory for Brain-Gut Axis Studies (LaBGAS),Translational Research Centre for Gastrointestinal Disorders (TARGID), Department of Chronic Diseases & Metabolism (CHROMETA), KU Leuven, Leuven, Belgium

**Keywords:** Irritable Bowel Syndrome, Clinical Protocols, Randomized Controlled Trial, Functional bowel disorders, Adult gastroenterology

## Abstract

**Introduction:**

Irritable bowel syndrome (IBS) is a disorder of gut-brain interaction characterised by abdominal pain and altered bowel habits and is associated with reduced quality of life. Although several treatments are effective at the group level, treatment responses vary substantially between individuals, highlighting the need for personalised approaches. This trial investigates moderators and mediators of treatment response to two established IBS interventions to determine for whom treatments are most effective and how they exert their effects.

**Methods and analysis:**

This parallel-group randomised controlled trial will enrol 235 adults with IBS from community, primary and tertiary care across Australia and the US. Participants will be randomised to a 12 week online fermentable oligosaccharides, disaccharides, monosaccharides and polyols (FODMAP) diet intervention targeting reduction of fermentable carbohydrate intake or an exposure-based cognitive behavioural therapy (E-CBT) programme targeting symptom-related avoidance behaviours, each with therapist support (dietitian or psychologist, respectively). The primary objective is to assess moderators and mediators of treatment response on gastrointestinal symptom severity, measured using the Gastrointestinal Symptom Rating Scale (GSRS-IBS), assessed weekly from baseline to week 12. Pre-specified moderators and mediators include gastrointestinal-specific anxiety and avoidance behaviour and total FODMAP intake; treatment preference will also be examined as a moderator. Treatment expectancy and credibility and therapeutic alliance will be assessed as covariates accounting for non-specific treatment effects. Secondary outcomes, assessed at baseline, post-treatment (12 weeks) and 3- and 6-month post-intervention follow-up, include IBS-specific symptom severity (measured using the IBS-Symptom Severity Scale, IBS-SSS), IBS-related quality of life, eating-related symptoms, psychological symptoms, extraintestinal symptom distress, stool frequency and consistency and gut microbiome composition and function. We will also assess gastrointestinal symptom severity by the GSRS-IBS at 3- and 6-month follow-up to assess maintenance. Linear mixed-effects models will assess treatment effects and moderation. Latent growth curve modelling and time-lagged mixed-effects models will examine mediation.

**Ethics and dissemination:**

Ethical approval was obtained from the University of Melbourne Human Research Ethics Committee (Project ID: 31186). This manuscript is based on protocol V.4.0 (06–04-26). Recruitment is ongoing. Results will be disseminated via peer-reviewed publications and presentations at scientific meetings.

**Trial registration number:**

NCT06940596.

**STRENGTHS AND LIMITATIONS OF THIS STUDY**

- This trial is designed with pre-specified moderators and mediators as primary analytical aims, enabling rigorous evaluation of treatment mechanisms and individual variability in treatment response in irritable bowel syndrome (IBS)—moving beyond group-level efficacy comparisons.
- The inclusion of two mechanistically distinct but efficacious interventions (fermentable oligosaccharides, disaccharides, monosaccharides and polyols diet and exposure-based cognitive behavioural therapy) provides an optimal comparative framework for evaluating treatment-specific pathways and identifying for whom each approach is most effective.
- Repeated assessment of symptoms and key psychological, dietary and microbiome variables throughout the intervention period enables examination of dynamic temporal relationships underlying treatment response.
- Interventions are delivered online with integrated therapist support, using a model informed by prior feasibility and acceptability testing, addressing well-recognised access barriers to specialised IBS care.
- Participant and therapist masking is not feasible given the nature of the interventions; however, outcome data will be analysed using de-identified study IDs masked to allocation, with treatment expectancy, credibility and alliance measured and adjusted for as covariates.

## Introduction

 Irritable bowel syndrome (IBS) is a disorder of gut-brain interaction which by the Rome IV criteria is characterised by recurrent abdominal pain associated with defecation or change in bowel habits, with a global prevalence of approximately 4%.^1^ IBS is associated with reduced quality of life^2^ and substantial healthcare and societal burden.^3^ Its pathophysiology involves interactions between biological, psychological and social processes,^4 5^ and the relative contribution of these mechanisms varies between individuals. Although several treatments demonstrate efficacy, a substantial proportion of patients do not experience adequate benefit.^6^

Two evidence-based but conceptually opposite interventions for IBS are the low fermentable oligosaccharides, disaccharides, monosaccharides and polyols (FODMAP) diet, which aims to restrict foods hypothesised to provoke symptoms, and exposure-based cognitive-behavioural therapy (E-CBT), which aims to reduce avoidance of symptom-provoking stimuli.^6^ Both are supported by meta-analytical evidence^7 8^ and recommended in current management guidelines.^9–11^ Despite their efficacy, both have limitations: the FODMAP diet can be difficult to adhere to and may contribute to long-term food avoidance, impairing food-related quality of life and increasing disordered eating risk^12^; E-CBT is not universally acceptable and varies in components and delivery across studies.^7 11^ To date, these interventions have not been directly compared in IBS.^6^

As treatment response varies substantially between individuals,^6^ understanding *which* treatment works best *for whom* (moderators) and *how* each exerts its effects (mediators) is a critical gap.^13^ Existing predictor and moderator research in IBS has examined biological, psychological and clinical characteristics, including gut microbiota, metabolome, depression and anxiety, dietary intake and gastrointestinal and extraintestinal symptoms; however, findings remain inconsistent, without reliable indicators for treatment selection.^14 15^ Within E-CBT, gastrointestinal-specific anxiety and behavioural avoidance have emerged as promising mediators and moderators^14^; for the low FODMAP diet, we found that gastrointestinal-specific anxiety, treatment expectancy and illness perceptions predict response.^16^ No trial has directly compared the two interventions while concurrently assessing these processes, and non-specific predictors such as treatment expectations and credibility, illness perceptions^16 17^ and therapeutic alliance^18^ may additionally shape outcomes.

Given IBS heterogeneity and the contrasting mechanisms of these treatments, trials directly comparing interventions while evaluating moderators and mediators of treatment response are needed. This randomised controlled trial (the exclude or expose in IBS Study) aims to investigate pre-specified moderators and mediators of treatment response to an internet-delivered FODMAP diet or E-CBT intervention. We illustrate the hypothesised relationships in figure 1.

**Figure 1:**
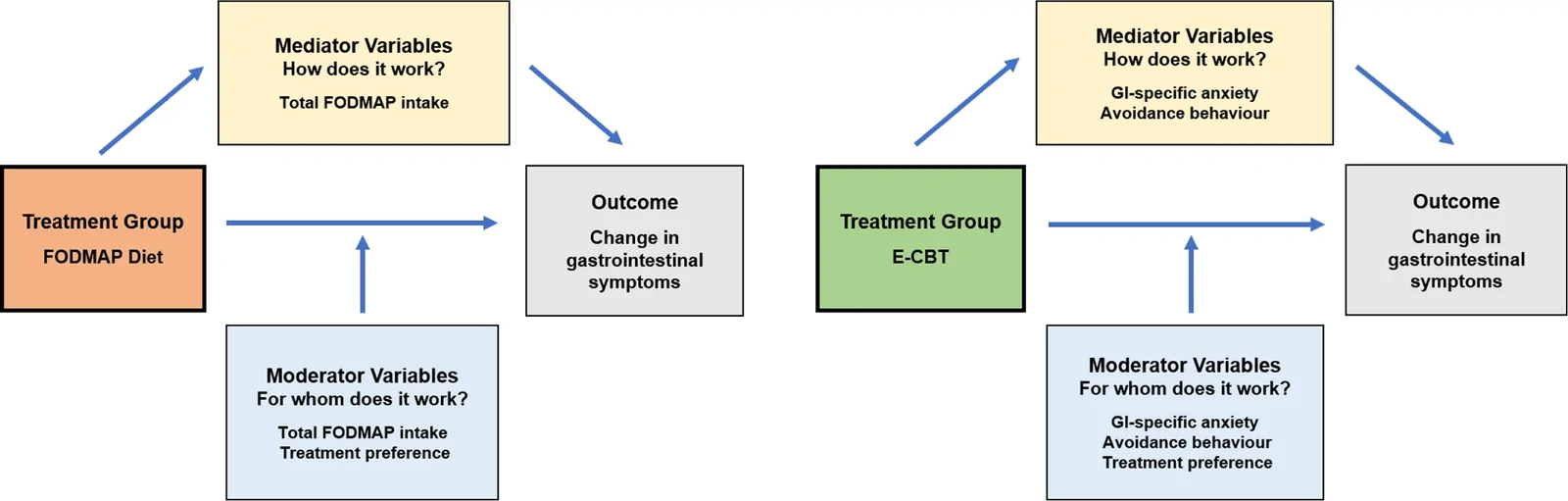
Conceptual model of treatment effects, moderators and mediators.

Conceptual model illustrating treatment effects of the FODMAP diet and E-CBT on IBS symptoms, including hypothesised baseline moderator and mediator pathways of treatment response. E-CBT, exposure-based cognitive behavioural therapy; FODMAP, fermentable oligosaccharides, disaccharides, monosaccharides and polyols; IBS, irritable bowel syndrome.

## Study hypotheses

### Primary hypothesis

No a priori difference in gastrointestinal symptom reduction is hypothesised between the FODMAP diet and E-CBT; both interventions are expected to produce clinically meaningful improvement. Bayesian analyses will quantify evidence for the null hypothesis of equivalent treatment effect.

### Moderator hypotheses

The following baseline variables will moderate treatment response such that:

- Higher baseline FODMAP intake will be associated with greater symptom improvement in the FODMAP diet compared with E-CBT.
- Higher baseline gastrointestinal-specific anxiety and avoidance behaviour will be associated with greater symptom improvement in E-CBT compared with the FODMAP diet.
- Allocation to preferred treatment will be associated with greater symptom improvement.

### Mediator hypotheses

The following mediation hypotheses will be tested:

- Reduction in FODMAP intake will mediate symptom improvement in the FODMAP diet condition but not in E-CBT.
- Reduction in gastrointestinal-specific anxiety and avoidance behaviour will mediate symptom improvement in the E-CBT condition but not in the FODMAP diet.

## Methods

### Study objectives, outcomes and hypotheses

#### Objectives

The primary objective is to assess moderators and mediators of treatment response to the FODMAP diet and E-CBT on gastrointestinal symptom severity over a 12-week intervention period.

Secondary objectives are to:

- Evaluate between- and within-group effects of the interventions on gastrointestinal symptoms and other clinical outcomes at 12 weeks and 3- and 6-month follow-up.
- Determine the proportion of responders, defined as participants achieving a minimally clinically important change (MCIC) in gastrointestinal symptom outcomes after intervention.
- Assess participant satisfaction, intervention acceptability, perceived mechanisms and post-intervention maintenance of dietary or behavioural strategies.

#### Moderator and mediator variables

The primary outcome variable is gastrointestinal symptom severity, measured using the Gastrointestinal Symptom Rating Scale-IBS (GSRS-IBS) total score. GSRS-IBS slopes, derived from weekly assessments between baseline and week 12, will be compared between intervention groups within the moderator and mediator models.

Moderator variables will be assessed at baseline; mediator variables will be assessed at baseline and repeatedly throughout the intervention and follow-up to enable examination of temporal relationships with treatment response. Pre-specified moderators and mediators include gastrointestinal-specific anxiety and avoidance behaviour and total FODMAP intake; treatment preference is additionally included as a moderator (table 1; online supplemental material 1). These variables were selected based on the conceptual opposition between the interventions^6^ and prior predictors of dietary^16^ and behavioural treatment response.^14^

**Table 1:** Clinical outcome, moderator, mediator and process variables and timepoints

Variable type	Variable (construct)	Measure (instrument)	Timepoints
Clinical outcome (pre-specified)	Gastrointestinal symptom severity	GSRS-IBS	Baseline; weekly during intervention (weeks 0–12)
Moderator (pre-specified)	Gastrointestinal-specific anxiety	VSI	Baseline
	Gastrointestinal-specific avoidance behaviour	IBS-BRQ	Baseline
	Total FODMAP intake	3-day food diary	Baseline
	Treatment preference	Single-item measure	Baseline
Moderator (exploratory)	IBS-related quality of life	IBS-QOL	Baseline
	ARFID symptoms	NIAS	Baseline
	Food-related fears	FFQ	Baseline
	Extraintestinal symptom distress	PHQ-12*	Baseline
	Depression severity	PHQ-9	Baseline
	Anxiety symptom severity	GAD-7	Baseline
	IBS symptom severity	GSRS-IBS	Baseline
	IBS subtype	7-day stool diary; BSFS	Baseline
	Gut microbiome composition and function	Stool sample†	Baseline
Mediator (pre-specified)	Gastrointestinal-specific anxiety	VSI	Baseline; 2-weekly; 12 weeks; follow-up
	Gastrointestinal-specific avoidance behaviour	IBS-BRQ	Baseline; 2-weekly; 12 weeks; follow-up
	Total FODMAP intake	3-day food diary	Baseline; 2-weekly; 12 weeks; follow-up
Mediator (exploratory)	Gut microbiome composition and function	Stool sample^†^	Baseline; 2-weekly; 12 weeks; follow-up
	Individual FODMAP components	3-day food diary	Baseline; 2-weekly; 12 weeks; follow-up
Process variables (for sensitivity analysis)	Treatment expectancy and credibility	CEQ	End of week 2
	Therapeutic alliance	WAI	End of week 2

Follow-up, 3 and 6 months after completion of the 12-week intervention.

* Derived from PHQ-15, Patient Health Questionnaire-15.

† Australian participants only.

BSFS, Bristol Stool Form Scale; CEQ, Credibility and Expectancy Questionnaire; FFQ, Fear of Food Questionnaire; FODMAP, fermentable oligosaccharides, disaccharides, monosaccharides and polyols; GAD-7, General Anxiety Disorder-7; GSRS-IBS, Gastrointestinal Symptom Rating Scale-IBS ; IBS, irritable bowel syndrome; IBS-BRQ, Irritable Bowel Syndrome-Behavioural Responses Questionnaire; IBS-QOL, Irritable Bowel Syndrome-Quality Of Life; NIAS, Nine Item Avoidant Restrictive Food Intake Disorder (ARFID) Screen ; PHQ-9, Patient Health Questionnaire-929; PHQ- 12, Patient Health Questionnaire-12; VSI, Visceral Sensitivity Index; WAI, Working Alliance Inventory.

Exploratory moderator and mediator variables include additional psychological, clinical, dietary and microbiome-related factors, assessed at baseline and longitudinally. Treatment-related process variables, including treatment expectancy, credibility and therapeutic alliance, will be assessed at the end of week 2 and included as covariates in sensitivity analyses accounting for non-specific (common) treatment factors.

#### Secondary outcome variables

Secondary outcomes include the IBS-SSS, IBS-related quality of life, eating-related symptoms, psychological symptoms, extraintestinal symptom distress and stool characteristics, all assessed as continuous variables with between-group comparisons at 12 weeks and 3- and 6-month follow-up, including maintenance of gastrointestinal symptom improvement (GSRS-IBS) at 3- and 6 months. To descriptively report clinically significant response rates, we will report the proportion of participants within each group achieving a ≥30% reduction in GSRS-IBS score^19^ and a ≥50-point reduction in IBS-SSS^20^ following treatment (table 2). See online supplemental material 1) for detail on all measures.

**Table 2:** Secondary outcomes and timepoints

Outcome (construct)	Measure (instrument)	Timepoints
Gastrointestinal symptom severity (3- and 6-month follow-up)	GSRS-IBS	Baseline and 12 weeks* (for MCIC threshold); 3 and 6 months
Gastrointestinal symptoms	IBS-SSS	Baseline; 12 weeks*; 3 and 6 months
IBS-related quality of life	IBS-QOL	Baseline; 12 weeks; 3 and 6 months
ARFID symptoms	NIAS	Baseline; 12 weeks; 3 and 6 months
Food-related fears	FFQ	Baseline; 12 weeks; 3 and 6 months
Extraintestinal symptom distress	PHQ-12^†^	Baseline; 12 weeks; 3 and 6 months
Depression severity	PHQ-9	Baseline; 12 weeks; 3 and 6 months
Anxiety symptom severity	GAD-7	Baseline; 12 weeks; 3 and 6 months
Stool frequency and consistency	7-day stool diary; BSFS	Baseline; 12 weeks; 3 and 6 months

* We will also report the proportion of participants achieving minimum-clinically important change (MCIC) thresholds on the GSRS-IBS (≥30% reduction) and IBS-SSS (≥50-point reduction) following treatment (12 weeks). 3 and 6 months, 3 and 6 months after completion of the 12 week intervention.

† Derived from PHQ-15, Patient Health Questionnaire-15.

ARFID, Avoidant Restrictive Food Intake Disorder; BSFS, Bristol Stool Form Scale.; FFQ, Fear of Food Questionnaire; GAD-7, General Anxiety Disorder-7; GSRS-IBS, Gastrointestinal Symptom Rating Scale-IBS ; IBS, irritable bowel syndrome; IBS-QOL, Irritable Bowel Syndrome-Quality of Life; IBS-SSS, Irritable Bowel Syndrome-Symptom Severity Score; NIAS, Nine Item Avoidant Restrictive Food Intake Disorder (ARFID) Screen ; PHQ-9, Patient Health Questionnaire-929; PHQ-12, Patient Health Questionnaire-12.

#### Study design and setting

This is a parallel, two-arm randomised controlled trial comparing two 12-week online treatment interventions for IBS delivered with asynchronous therapist support. Following baseline assessments, eligible participants will be randomised 1:1 to receive either the online FODMAP diet or online E-CBT intervention. Follow-up assessments will be conducted at 3 and 6 months after completion of the 12-week intervention. The trial is being conducted across Australia and the United States (US).

The trial was prospectively registered with ClinicalTrials.gov (NCT06940596); refer to the registry for all WHO Trial Registration Data Set items, trial protocol and statistical analysis plan. The study flow is shown in figure 2, and the Standard Protocol Items: Recommendations for Intervention Trials (SPIRIT) checklist^21^ is provided in online supplemental material 2).

**Figure 2:**
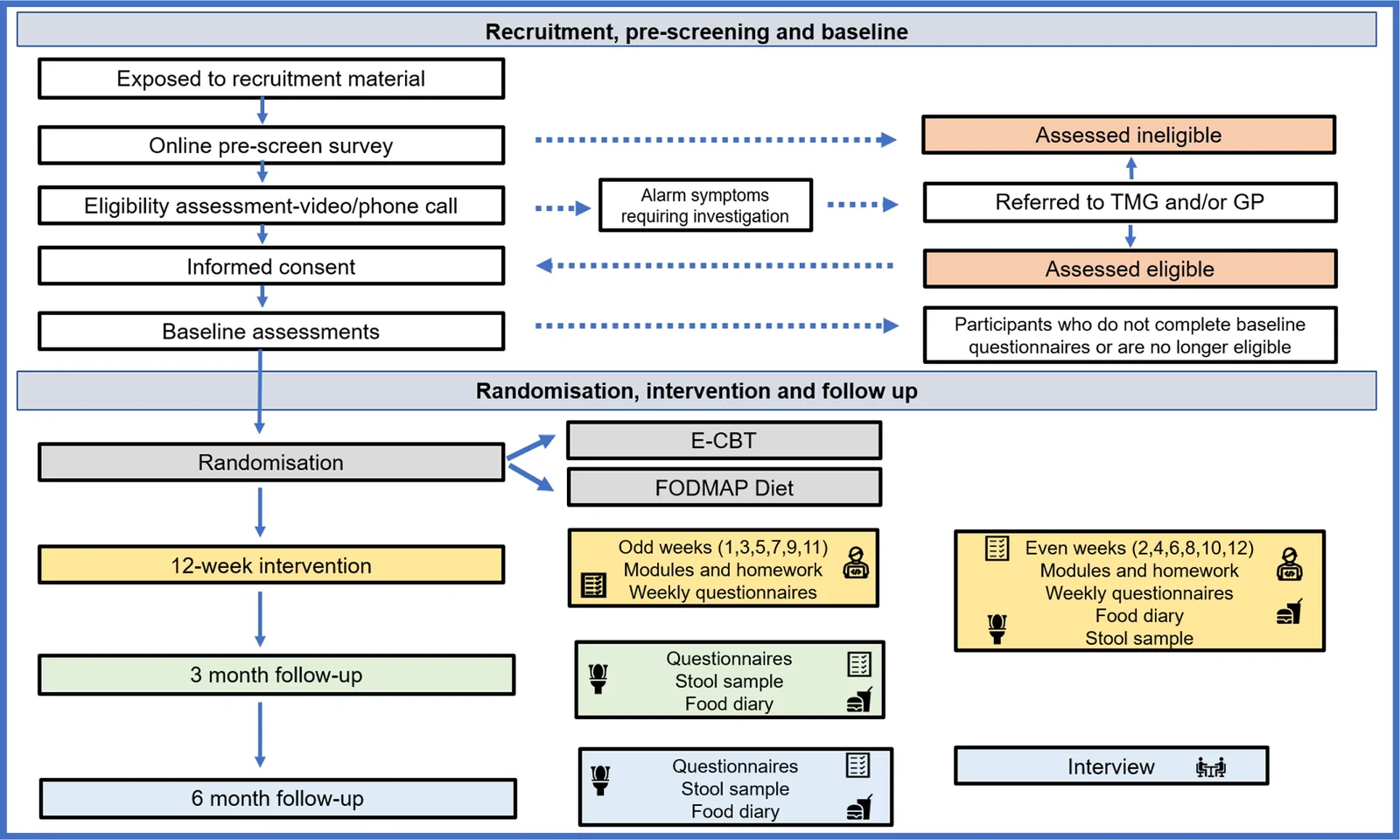
Study flow.

Alarm symptoms: blood in stool, recent unexplained/unintentional weight loss >5% body weight, onset of gastrointestinal symptoms >50 years, family history of gastrointestinal diseases or cancers, large volumes of diarrhoea at night, fever associated with gastrointestinal symptoms, recurrent vomiting, persistent unexplained iron deficiency. E-CBT, exposure-based cognitive behavioural therapy; FODMAP, fermentable oligosaccharides, disaccharides, monosaccharides and polyols; TMG, Trial Management Group.

### Participants

#### Eligibility criteria

Adults ≥18 years with a clinician-confirmed diagnosis of IBS meeting Rome IV criteria, symptomatic at time of randomisation (IBS-SSS≥175) and resident in Australia or the US are eligible. Key exclusions include pregnancy, other gastrointestinal conditions (eg, inflammatory bowel disease, coeliac disease), active eating disorder (Eating Disorder Examination-Questionnaire Short Form-8≥4), and severe depressive symptoms (Patient Health Questionnaire-9>20).^22^ Full eligibility criteria are provided in online supplemental material 3).

#### Recruitment and screening

Participants will be recruited via self-referral using multiple strategies, including online platforms and social media, and via clinician referral through clinical networks (online supplemental material 4). Recruitment materials direct potential participants to a Research Electronic Data Capture (REDCap; Vanderbilt University, Nashville, Tennessee, US) landing page containing study information and a pre-screening questionnaire (screening measures summarised in online supplemental material 1).

Potentially eligible participants are invited to a screening interview with a trained member of the study team to confirm eligibility; where indicated, participants may be asked to obtain medical clearance from their treating clinician. Eligible participants provide electronic informed consent, including provisions for the collection and use of stool specimens, and are assigned a programme start date and scheduled for baseline assessment.

#### Baseline assessments

Baseline assessments are completed prior to randomisation to confirm eligibility and establish pre-intervention measures. Participants must have an IBS-SSS score ≥175 within the week prior to programme commencement to proceed to randomisation. Baseline data include a 7-day stool diary, 3-day food diary, stool sample (Australian participants only, reflecting international shipping infeasibility and resource constraints limiting collection to a subset of participants) and self-report questionnaires assessing moderator, mediator and process variables (table 3; online supplemental material 1. Baseline data are valid for 1 month.

**Table 3:** Schedule of activities and assessments

Activity/assessment	Pre-screening online	Enrolment video call	Baseline online	Randomisation	12-week intervention*												Follow-up	
Timepoint		T1	T2	T3	*T4*	*T5*	T6	*T7*	*T8*	*T9*	*T10*	*T11*	*T12*	*T13*	*T14*	*T15*	*T16*	*T17*
Days		> −13	−13	-2	0	7	14	21	28	35	42	49	56	63	70	77		
Pre-screening survey	X																	
Rome IV	X																	
IBS-SSS	X		X													X	X	X
EDE-Q8	X																	
PHQ-9	X		X													X	X	X
Informed consent		X																
7-day stool diary			X													X	X	X
3-day food diary			X			X		X		X		X		X		X	X	X
Sociodemographic questionnaire			X															
GSRS-IBS			X		X	X	X	X	X	X	X	X	X	X	X	X	X	X
VSI			X			X		X		X		X		X		X	X	X
IBS-BRQ			X			X		X		X		X		X		X	X	X
IBS-QOL			X													X	X	X
NIAS			X													X	X	X
FFQ			X													X	X	X
PHQ-12^†^			X													X	X	X
GAD-7			X													X	X	X
Body weight			X													X	X	X
Stool sample			X			X		X		X		X		X		X	X	X
Randomisation				X														
Participants register for online programme				X														
Participants follow the 12-week intervention					X	X	X	X	X	X	X	X	X	X	X	X		
CEQ						X												
WAI						X												
Protocol compliance questionnaire‡					X	X	X	X	X	X	X	X	X	X	X	X	X	X
Adverse event questionnaire							X			X			X			X	X	X
CSQ																X	X	X
Programme acceptability questionnaire																X		
Post intervention behaviour questionnaire																	X	X

* 12-week intervention, participants randomised to either the FODMAP diet programme or the exposure-based CBT programme.

† PHQ-12 will be derived from PHQ-15 questionnaire.

‡ *Protocol compliance measure of new medications or treatments.

BSFS, Bristol Stool Form Scale; CEQ, Credibility and Expectancy Questionnaire; CSQ, Client Satisfaction Questionnaire; EDE-Q8, Eating Disorder Examination-Questionnaire Short Form 8; FFQ, Fear of Food Questionnaire; GAD-7, General Anxiety Disorder-7; GSRS-IBS, Gastrointestinal Symptom Rating Scale-Irritable Bowel Syndrome ; IBS-BRQ, Irritable Bowel Syndrome-Behavioural Responses Questionnaire; IBS-QOL, Irritable Bowel Syndrome-Quality Of Life; IBS-SSS, Irritable Bowel Syndrome-Symptom Severity Score; NIAS, Nine Item Avoidant Restrictive Food Intake Disorder (ARFID) Screen ; PHQ-9, Patient Health Questionnaire-929; PHQ-12, Patient Health Questionnaire-12; PHQ-15, Patient Health Questionnaire-1521; VSI, Visceral Sensitivity Index; WAI, Working Alliance Inventory.

#### Randomisation and masking

The allocation sequence was generated using an external randomisation tool (https://www.sealedenvelope.com/simple-randomiser/) by a member of the research team not involved in intervention delivery and uploaded directly to the REDCap randomisation module; the sequence file was not accessible to the study team following upload, and staff could not foresee upcoming assignments. Participants are randomised 1:1 following completion of baseline assessments, stratified by country (Australia or United States) to balance intervention allocation within each country, using blocks of 10. Recruitment to date indicates Australian participants will comprise a larger proportion of the final sample reflecting greater reach of recruitment advertising and referral networks in Australia to date; however, equal representation across countries is not required for the planned analyses.

Due to the nature of the interventions, participants, therapists and study personnel cannot be masked to allocation. To minimise analytic bias, data analysis will be conducted using de-identified study identifications that do not indicate treatment group, and intervention-specific measures (eg, compliance) will be calculated external to the main analytic dataset to avoid inadvertent unmasking of the analyses.

#### Trial procedures and follow-up

During the 12-week intervention, participants complete weekly questionnaires and fortnightly 3-day food diaries via REDCap, with automated reminders to support completion (online supplemental material 5). A subset of Australian participants (n=100) will provide stool samples fortnightly for gut microbiome analysis, an exploratory moderator and mediator of treatment response (see sample size estimation). Follow-up assessments are conducted at 3- and 6-month post-intervention and include questionnaires, a 7-day stool diary, a 3-day food diary and a stool sample (Australian microbiome sub-sample only). Participants have 1 week to complete assessments, with two reminder notifications provided. During follow-up, participants may contact the study team with intervention-related questions; new IBS-related therapies are recorded via the protocol compliance questionnaire but are not considered protocol deviations. All participants will be invited to participate in a semi-structured interview at 6-month follow-up to explore intervention experience and acceptability, perceived mechanisms and follow-up use of intervention strategies. The study flow is summarised in figure 2; the schedule of assessments is presented in table 3.

### Interventions

#### Overview

Participants are randomised to one of two 12-week online interventions—the low FODMAP diet or E-CBT—both delivered via the Canvas e-learning platform (Instructure; Salt Lake City, Utah, USA). Participants complete modules sequentially and submit weekly homework tasks for therapist review and feedback. Throughout, ‘therapist’ refers to the treating clinician in each arm: an Accredited Practising Dietitian (FODMAP diet) or a Clinical Psychologist (E-CBT). All therapists are clinicians registered with the Australian Health Practitioner Regulation Agency and deliver asynchronous support across both countries. The two interventions are matched for content length, therapist contact time (approximately 15 min per participant per week) and expected participant engagement (approximately 1–2 hours per week). An introductory module orients participants to Canvas, study expectations and therapist interaction prior to intervention commencement.

#### FODMAP diet intervention

The online FODMAP intervention is a 12-week dietitian-supported programme developed by the research team using an evidence-, theory- and person-based approach (Phase I development study^23^; Phase II feasibility, acceptability and proof-of-concept study).^24^ The FODMAP diet is a three-phase approach: Phase 1 restriction, limiting foods high or moderate in FODMAPs to assess symptom response; Phase 2 reintroduction, systematic food challenges to identify individual FODMAP tolerance and Phase 3 personalisation, long-term dietary adaptation based on individual responses.

Content is delivered across 12 weekly modules with support from an Accredited Practising Dietitian. Early modules introduce IBS, the FODMAP diet rationale and preparation for the restriction phase; subsequent modules guide participants through structured restriction, reintroduction challenges and personalisation of the diet. A detailed module schedule and content overview are provided in online supplemental material 6).

#### Exposure-based cognitive behavioural therapy intervention

The E-CBT programme is an internet-delivered adaptation of an established intervention developed for IBS and evaluated in pilot and randomised trials.^25–27^ The original Swedish-language programme was translated into English and adapted for delivery via Canvas by the research team.

The intervention comprises six sequential steps delivered over 12 weeks with support from a Clinical Psychologist. Early modules introduce the cognitive-behavioural model of IBS,^25–27^ a self-observation strategy and psychoeducation in preparation for behavioural changes. The core component—exposure-based behavioural change—commences from week 5 and includes (i) exposure to symptom-provoking stimuli, (ii) reduction of behaviours aimed at controlling or avoiding symptoms and (iii) engagement in previously avoided situations. Participants submit weekly ‘exposure reports’ documenting their behavioural exercises and progress. Final modules focus on consolidation of skills and relapse prevention. A detailed module schedule is provided in online supplemental material 7).

#### Therapist support and fidelity

Therapists will respond to participant homework submissions and messages within two working days, guided by intervention-specific response message banks that provide clarification, practical guidance and encouragement, developed by the study team to support consistency.

At least two dietitians and two psychologists will be active at any time to mitigate clinician bias and ensure continuity of support. Therapists are trained and supervised by the senior clinicians who developed the FODMAP diet programme (JA, CKY) and E-CBT programme (BL, HBM), respectively. Fidelity is monitored via a dedicated clinician communication channel for case discussion and consistency checks, and monthly review of a random sample of therapist responses.

#### Participant adherence and engagement

Adherence to both interventions will be assessed via self-reported completion of core components in weekly homework submissions and, for the FODMAP diet intervention, objective dietary intake from 3-day food diaries. Adherence criteria are pre-specified by intervention phase (table 4; online supplemental material 8) and reported descriptively as an exploratory outcome.

**Table 4:** Summary of adherence

FODMAP diet		
Intervention phase	Assessment method	Adherence definition
Restriction (weeks 3–6)	Weekly homework reports; 4-point Likert scale adherence question	Adherent if ≥50% adherence (‘frequently/always’) on ≥2 of 4 weeks
	3-day food diary (week 6)	Adherent if ≤12 g/day or ≥30% reduction from baseline
Reintroduction (weeks 7–12)	Weekly homework reports; food challenges undertaken	Adherent if ≥1 food re-challenge as per challenge guidelines per week
Personalisation (follow-up 3 and 6 months)	Self-report question reflecting current diet practice	Descriptive classification (strict, personalised, partial, habitual)

E-CBT		
Intervention phase	Assessment method	Adherence definition
Self-observation (weeks 1–4)	Weekly homework reports; self-observation activities undertaken	Adherent if ≥1 exercise/week
Exposure (weeks 5–12)	Weekly homework exposure reports	Adherent if ≥1 exposure/week for ≥5 weeks

E-CBT, exposure-based cognitive behavioural therapy; FODMAP, fermentable oligosaccharides, disaccharides, monosaccharides and polyols.

Engagement will be tracked via Canvas analytics, including module access and homework completion (at least one module-specific response weekly; for E-CBT, at least one exposure report weekly for weeks 5–12), recorded by therapists in REDCap.

#### Data collection and outcome measures

Data will be collected using validated patient-reported, psychological, dietary, clinical and acceptability measures (tables 1 and 2) throughout the intervention and follow-up periods to assess clinical outcomes, moderator and mediator variables and process variables. Assessment timepoints are summarised in table 3; detailed instrument descriptions are provided in online supplemental material 1).

Intervention acceptability and participant satisfaction will be assessed at end of intervention using the Client Satisfaction Questionnaire,^28 29^ a brief programme acceptability questionnaire and a post-intervention behaviour questionnaire administered at follow-up. Safety and protocol compliance will be monitored via adverse events (AEs) and protocol compliance questionnaires (see Safety monitoring and reporting).

#### Participant retention and withdrawal

Participants who discontinue the intervention will be encouraged to continue weekly and follow-up assessments. Participants who do not complete key timepoints and fail to re-engage will be classified as lost to follow-up at the 12-week, 3- or 6-month timepoint. Participants who withdraw receive no further contact. Data collected prior to withdrawal will be retained and included in analyses unless the participant specifically requests data removal.

#### Protocol deviations

Participants are asked to maintain baseline treatments (medications, supplements, therapies) throughout the 12-week intervention where possible. New treatments are recorded weekly via the protocol compliance questionnaire and reviewed by the study team. During the intervention, commencing a new (non-study) dietitian-led IBS restriction diet or clinician-delivered IBS behavioural therapy is considered a protocol deviation that may interfere with the allocated intervention and may result in discontinuation, as determined by the Trial Management Group (TMG); these participants will be encouraged to continue study assessments. Other new treatments that do not affect intervention participation are recorded as minor protocol deviations and reviewed at team meetings. New treatments commenced during the 3- and 6-month follow-up are recorded but are not considered protocol deviations.

### Statistical methods

#### Sample size estimation

Due to the complexity of the proposed analyses, including parallel process latent growth curve modelling for mediation and mixed-effects models for moderation, formal analytic power calculations are not straightforward. Simulation studies of comparable clinical trials^30–32^ suggest that approximately 200 participants are sufficient to detect meaningful mediation effects; this sample size is also considered adequate for moderation analyses, which involve fewer parameters.^13^

Although no between-group difference in treatment effect is hypothesised, we performed a simulation to estimate the power to detect statistically significant differences between the groups. We used the observed weekly means, covariance and missingness pattern of GSRS-IBS ratings from 153 patients who received E-CBT in a previous study^33^ to simulate the present study (n=200; 1000 replications), analysed with linear mixed models including a random intercept and a random slope for time. The present study provides 80% power to detect a small-to-moderate between-group difference in change of Cohen’s d=0.44 (standardised on the baseline SD), favouring either E-CBT or FODMAP.

Allowing for 15% attrition at the week-12 primary outcome assessment, 235 participants will be randomised. If attrition exceeds expectations, recruitment may be extended up to a maximum of 285 participants (30% attrition threshold).

The microbiome sub-sample (n=100 Australian participants; see Baseline assessments) reflects feasibility and resource constraints rather than a formal power calculation, consistent with its status as an exploratory outcome.

#### Population to be analysed

Analyses will follow the intention-to-treat principle, including all randomised participants with at least one outcome measurement. A per-protocol sensitivity analysis will include participants meeting pre-defined adherence criteria for their allocated intervention (online supplemental material 8).

#### Handling of missing data

Models will use all available data to estimate parameters and standard errors without imputation. Missing data will be handled using full information maximum likelihood estimation under the missing at random assumption.^34 35^ Missing data patterns will be visualised and described before primary analyses. If more than 20% of participants do not contribute at least two data points for the primary outcome (GSRS-IBS), or more than 20% of data are missing at 12 weeks for secondary outcomes, multiple imputation will be conducted as a sensitivity analysis to assess robustness to missing data assumptions.

#### Methods of analysis

Statistical analysis will be conducted using R (R Foundation for Statistical Computing; Vienna, Austria) and Mplus (Muthén & Muthén; Los Angeles, California, USA). Baseline characteristics will be summarised descriptively by intervention group: mean±SD or median (IQR) for continuous variables and n (%) for categorical variables. Distributional assumptions will be examined prior to analysis. Before primary outcome, mediation and moderation analysis are performed, we will select the growth shape (linear, exponential time, log or square-root) using an unconditional model across both groups (with only time as predictor) based on the Akaike Information Criterion. Significance is set at p<0.05 for frequentist analyses. Given the a priori hypothesis of no between-intervention difference in primary outcome, Bayesian analyses will complement frequentist analyses to evaluate evidence in favour of the null hypothesis. The proportion of responders (participants achieving a MCIC) will be reported descriptively with 95% CIs.

Country is included as a covariate in primary and secondary analyses, with sensitivity analyses examining consistency across country strata. Unadjusted p values will be reported together with false-discovery rate (FDR) adjusted p values (using the Benjamini-Hochberg procedure). FDR correction will be applied within each class of analyses, ie, secondary outcome, moderation, mediation, microbiota and dietary intake analyses. No FDR correction will be applied to the primary outcome analysis. Treatment expectancy, credibility and therapeutic alliance will be entered as covariates in sensitivity analyses to evaluate robustness of between-group effects to non-specific (common) treatment factors.

#### Primary outcome analyses

A linear mixed-effects model with random intercept will assess between-group differences in change in GSRS-IBS (total score) over time (from baseline to week 12), with group×time interaction as the primary test of interest.

#### Secondary outcome analyses

Linear mixed-effects models will assess between-group differences in secondary outcomes (total scores) at week 12 and at 3- and 6-month follow-up. In these analyses, time will be entered as a categorical predictor with baseline as the reference value. Within-group maintenance of effects from end-of-intervention to follow-up will also be examined.

#### Moderation analyses

Moderation analyses will use linear mixed-effects models with three-way interaction terms (group×time×moderator) predicting GSRS-IBS scores over the 12-week measurement period.

#### Mediation analysis

Mediation analyses are designed to test pre-specified mediation hypotheses over the 12-week measurement period. Parallel process latent growth curve modelling will assess between-group mediation effects, and random intercept cross-lagged panel models (RI-CLPM) will assess within-group time-lagged associations. For both analyses, differential mechanisms across treatments will be considered confirmed based on pre-specified, opposite-direction patterns of mediator-outcome effects for the FODMAP-specific (FODMAP intake) versus E-CBT-specific (gastrointestinal-specific anxiety, avoidance behaviour) mediators; full confirmatory criteria are provided in online supplemental material 9). The within-group RI-CLPM analyses are considered exploratory and are intended to complement the between-group mediation analyses by characterising the temporal pattern of mediator-outcome associations within each treatment. We do not formally test between-group differences in cross-lagged coefficients.

#### Microbiota analyses

Stool samples from the Australian microbiome sub-sample (n=100) will be analysed using 16S rRNA sequencing and shotgun metagenomics to characterise microbiome composition and function. Analyses will include diversity metrics, differential abundance, functional pathway and multivariate associations with treatment response. Detailed laboratory and bioinformatics procedures are provided in online supplemental material 10).

#### Dietary intake analysis

Dietary intake data will be analysed using the Monash University FODMAP Calculator (Monash University; Melbourne, Australia) and FoodWorks (Xyris Software Pty, Ltd, Brisbane, Australia). Total FODMAP intake (g/day) is calculated as the sum of excess fructose (fructose minus glucose), lactose, fructans, galacto-oligosaccharides and polyols (mannitol and sorbitol), averaged across 3 day food diaries. Energy, macronutrient and fibre intake will also be assessed. A sensitivity analysis will compare dietary data collected via mobile application versus paper diary to assess measurement consistency. Detailed procedures are provided in online supplemental material 11.

#### Qualitative analysis

A nested qualitative study will explore participants’ experiences of the interventions. All participants will be invited to a semi-structured interview at 6-month follow-up conducted by a trained researcher. Interviews will broadly explore intervention experience and acceptability, perceived mechanisms of change and use of intervention strategies during follow-up. The interview guide and detailed procedures (including sampling) are under development, will be informed by emerging trial processes and co-investigator input and will be finalised and submitted for ethics approval before qualitative data collection commences. Interviews will be audio-recorded, transcribed verbatim, de-identified and analysed using reflexive thematic analysis^36 37^ supported by NVivo software (QSR International Pty Ltd, Melbourne, Victoria, Australia). Findings will be reported in line with the Consolidated Criteria for Reporting Qualitative Research (COREQ)^38^ and, where informative, integrated with quantitative outcomes to contextualise treatment response.

### Data management and security

Data will be collected and managed using REDCap (hosted on University of Melbourne secure infrastructure in Australia) for questionnaire and eligibility data, and Canvas for intervention delivery and engagement data. Data quality is maintained through REDCap validation rules at point of entry and scheduled data checks by the trial coordinator. Participants will be identified by study identification only; personal identifying information will be stored separately from study data in a restricted-access REDCap project, accessible only to the trial coordinator and nominated research team members. De-identified data will be retained long-term post-trial, per institutional policy, for potential use in future ethically approved research.

### Trial oversight and monitoring

The TMG, comprising the principal investigator (PI), trial coordinator, senior study clinicians (dietitian, psychologist), gastroenterologist and PhD candidate, will oversee trial conduct, including safety monitoring and protocol adherence. Day-to-day management will be conducted by a core sub-group of the TMG, at minimum the PI and trial coordinator. REDCap data and Canvas engagement records will be reviewed monthly for data quality, completeness and protocol adherence. No interim efficacy analyses or formal stopping rules are planned; the TMG may recommend halting the trial in response to emergent safety concerns.

### Safety monitoring and reporting

AEs will be assessed every 3 weeks during the intervention and at 3- and 6 month follow-up (table 3; online supplemental material 1). The TMG will assess each AE for severity (mild, moderate or severe), seriousness and relatedness to the intervention (definitions provided in online supplemental material 12). Serious adverse events related to the intervention will be reported to the University of Melbourne Human Research Ethics Committee and sponsor within required timeframes.

Participants will retain access to their usual clinical care throughout the trial and at its conclusion; no additional post-trial care will be provided by the study team. Intervention-related AEs will be managed as described above, and participants will be advised to seek review from their usual clinician or emergency care where appropriate.

## Ethics and dissemination

### Research ethics approval

Ethical approval was obtained from the University of Melbourne Human Research Ethics Committee. Any protocol amendments will be submitted to the Committee for approval and communicated to participants as required.

### Dissemination

Findings will be disseminated via peer-reviewed publications, conference presentations and a lay summary provided to participants. Beginning 3 months following publication, de-identified participant data, the protocol, plain language statement and the statistical analysis plan will be made available to researchers from recognised institutions with ethically approved proposals under University of Melbourne data-sharing agreements.

### Patient and public involvement statement

This trial was informed by prior development and feasibility work with adults with IBS and clinicians (Phase I;^23^ Phase II).^24^ Patient representatives contributed to intervention design, study procedures and participant-facing materials. Patients were not involved in study conduct or analysis. Findings will be shared with participants via a plain language summary.

### Trial status

The first participant provided informed consent on 30 May 2025, and the first treatment group commenced on 16 June 2025. At the time of publication, the planned recruitment completion date is July 2027, with study completion (including 6-month follow-up) estimated for April 2028.

### Protocol version

This manuscript describes protocol V.4.0 (06-04-26). Protocol amendments are approved by the University of Melbourne Human Research Ethics Committee, recorded on ClinicalTrials.gov registry and communicated to trial participants when relevant. No amendments have altered the primary outcome or core study design. Protocol version history: V.1.0 (27-03-25, original approval); V.2.0 (29-05-25); V.3.0 (15-09-25); V.4.0 (06–03-26).

## Discussion

To our knowledge, this is the first trial to directly compare a dietary and a behavioural intervention for IBS with moderators and mediators pre-specified as primary aims. Strengths include weekly symptom and fortnightly mediator assessment, which enables temporal analyses; interventions matched for content length, therapist contact and expected engagement; a delivery model informed by prior development and feasibility work and recruitment across two countries and multiple care settings. Limitations include that participants and therapists cannot be masked to intervention allocation, and dietary intake and psychological mediators rely on self-report. As the trial compares two active interventions, it is designed to identify differential moderators and mediators of response rather than efficacy relative to no treatment; improvements common to both groups therefore cannot be attributed specifically to treatment. Microbiome sampling is restricted to an Australian sub-sample, which limits these exploratory analyses. The sample is expected to be weighted toward Australian participants, and people who self-select into an online trial may differ from the broader IBS population.

## Supplementary material

10.1136/bmjopen-2026-126664 online supplemental file 1
