# Supplementary material for: Exclude or expose in irritable bowel syndrome study: what works for whom and how? Protocol for a randomised controlled trial of internet-delivered FODMAP diet versus exposure-based cognitive behavioural therapy
Source: BMJ Open. 2026 Nov 11;16(11):e126664. doi: 10.1136/bmjopen-2026-126664 (PMC13756487; doi:10.1136/bmjopen-2026-126664)
Supplement: Supplementary file 1 [file bmjopen-16-11-s001.pdf]

EXCLUDE OR EXPOSE IN IRRITABLE BOWEL SYNDROME: SUPPLEMENTAL MATERIAL

Supplemental material 1: Screening and assessment measures

Supplemental material 2: Table S2 SPIRIT (Standard Protocol Items: Recommendations for Intervention Trials) checklist

Supplemental material 3: Box S3: Inclusion and exclusion criteria

Supplemental material 4: Box S4: Recruitment strategies

Supplemental material 5: Table S5: Access and reminder process schedule

Supplemental material 6: Table S6: Online FODMAP programme summary and schedule

Supplemental material 7: Table S7: Online E-CBT programme summary and schedule

Supplemental material 8: Assessment of participant adherence to online programmes

Supplemental material 9: Mediation analysis confirmatory criteria

Supplemental material 10: Microbiome data collection and analyses

Supplemental material 11: Dietary intake data collection and analyses

Supplemental material 12: Table S12: Safety monitoring and reporting definitions

SUPPLEMENTAL MATERIAL 1

Screening and assessment measures

Screening measures

Rome IV Irritable Bowel Sundrome (IBS) Screening Questionnaire: Used to identify likely cases of IBS based on Rome IV criteria, requiring recurrent abdominal pain (≥1 day/week over the previous 3 months) associated with at least two of the following: relation to defecation, change in stool frequency, or change in stool form[1,2].

IBS-Symptom Severity Score (IBS-SSS): A 5-item measure of IBS symptom severity (range 0–500), with higher scores indicating greater severity. A score ≥175 is required for eligibility[3].

The Eating Disorder Examination-Questionnaire Short Form 8 (EDE-Q8): An 8-item self-report measure of eating disorder psychopathology over the past 28 days, (range 0-6 for each item), with higher scores indicating greater symptom severity[4]. Participants scoring ≥4 are excluded to minimise inclusion of clinically significant eating disorders[5].

Patient Health Questionnaire-9 (PHQ-9): A 9-item measure of depressive symptoms (range 0–27), with higher scores indicating greater severity[6]. Participants scoring >20 are excluded from the study.

Baseline and intervention measures

Sociodemographic and clinical questionnaire: Collected at baseline, including age, gender, BMI (derived from self-reported height and weight), IBS history, education, employment, country of residence, ethnicity, medication use and comorbidities**.**

Stool diary (7-day): Assesses stool frequency and consistency using the Bristol Stool Form Scale (BSFS), and enables classification of IBS subtype according to Rome IV criteria[1,7].

Food diary (3-day): Participants record food intake over three days (including one weekend day). Data are used to estimate total and individual FODMAP intake (g/day). (See supplemental material 11).

Stool sample: Australian participants only, as international shipping of stool samples between the United States and Australia is not feasible. Collected using standardised kits to assess gut microbiome composition and function. (See supplemental material 10).

Gastrointestinal Symptom Rating Scale-IBS (GSRS-IBS): A 13-item measure of gastrointestinal symptom severity (range 13–91), with higher scores indicating greater severity[8]. Comprised of five subscales: pain, bloating, diarrhoea, constipation, and early satiety[9]. The GSRS-IBS has demonstrated good internal consistency (Cronbach's α 0.74–0.85) and test–retest reliability (.55 and .70)[8]. A symptom improvement ≥30% from baseline is considered clinically meaningful[10].

IBS-Symptom Severity Score (IBS-SSS): A 5-item measure of IBS symptom severity; frequency and severity of abdominal pain, severity of bloating, dissatisfaction with bowel habits and impact on life in general (range 0–500), with higher scores indicating greater severity. A ≥50-point reduction is considered clinically meaningful[3].

Visceral Sensitivity Index (VSI): A 15-item measure of gastrointestinal-specific anxiety (range 0–75), with higher scores indicating greater anxiety[11,12]. The VSI has demonstrated excellent internal consistency (Cronbach's α ≈0.93)[12].

Irritable Bowel Syndrome-Behavioural Responses Questionnaire (IBS-BRQ): A 26-item measure of IBS-related avoidance behaviours (range 26–182), with higher scores indicating greater avoidance[13]. The IBS-BRQ has demonstrated good internal consistency (Cronbach's α ≈0.86)[13].

Nine Item ARFID (Avoidant Restrictive food intake disorder) Screen (NIAS): A 9-item measure of avoidant restrictive food intake disorder symptoms (range 0–45), with higher scores indicating greater severity[14,15]. Comprised of three subscales: fear of adverse consequences, lack of interest/appetite, and sensory sensitivity.

Fear of Food Questionnaire (FFQ): An 18-item measure of food-related fears (range 0–90), with higher scores indicating greater fear and avoidance[16]. Comprised of five subscales: gastrointetinal fears, food fears, food avoidance, social impairment, and distress/loss of pleasure.

Irritable Bowel Syndrome-Quality of Life (IBS-QOL): A 34-item measure of IBS-related quality of life (range 0–100), with higher scores indicating better quality of life[17]. The IBS-QOL includes eight domains: dysphoric thoughts; interference with activity; body image; health worry; food avoidance; social reaction; sexual; relationship. The IBS-QOL shows high internal consistency (Cronbach's α ≈0.95)[17].

Patient Health Questionnaire-9 (PHQ-9): A 9-item measure of depressive symptoms (range 0–27), with higher scores indicating greater severity[6].

Generalised Anxiety Disorder-7 (GAD-7): A 7-item measure of anxiety severity (range 0–21), with higher scores indicating greater severity[18].

Patient Health Questionnaire-12 (PHQ-12): A modified version of the PHQ-15[19] excluding gastrointestinal items, assessing non-gastrointestinal somatic symptoms (range 0–22), with higher scores indicating greater extraintestinal symptom burden[20]. The menstruation-related item will be removed to simplify interpretation and avoid introducing gender-related bias in the scoring[20].

Treatment related measures

The Credibility/Expectancy Questionnaire (CEQ): A 6-item measure of treatment credibility and expectancy (range 6-52)[21].

The Working Alliance Inventory for Internet Interventions Questionnaire (WAI-I): A 12-item measure assessing therapeutic alliance in guided digital interventions, with higher scores indicating stronger alliance[22]. Comprised of two subscale dimensions: task and goal agreement with the programme; and bond with therapist.

Treatment Preference: Assessed using a single item measure of preferred intervention (FODMAP diet, CBT, or no preference)[23,24].

The Client Satisfaction Questionnaire: An 8-item measure of overall satisfaction with the intervention (range 8–32), with higher scores indicating greater satisfaction[25,26].

Programme acceptability questionnaire: A brief 3-item measure assessing perceived helpfulness and likelihood of recommending the programme, with optional qualitative feedback.

Post intervention behaviour questionnaire: Assessed at 3- and 6-month follow-up. Participants report their ongoing application of intervention strategies following treatment. For the FODMAP diet, participants select the response that best reflects their current dietary pattern: strict low FODMAP diet (restriction), reintroduction/personalisation (including tolerated high-FODMAP foods), partial adherence (~50% restriction), return to habitual diet, or other. For the E-CBT intervention, participants report their current use of behavioural

strategies: regularly performing exposure exercises, occasional use, no longer required, not helpful, or other.

Process and safety measures

Adverse Events: Assessed 3-weekly during 12-week intervention and at 3- and 6-months follow-up via self-report. Three questions asking about changes to health in the past 3 weeks including admission to hospital, any new diagnoses, and any other significant changes to health.

Protocol Compliance: Assessed via weekly self-report including initiation of medications, supplements (eg, probiotics), antibiotics, or other exclusionary treatments.

SUPPLEMENTAL MATERIAL 2

Table S2: SPIRIT CHECKLIST

Section / Topic	No	SPIRIT 2025 checklist item description	Reported on page no.
**Administrative information**			
Title and structured summary	1a	Title stating the trial design, population, and interventions, with identification as a protocol	1
	1b	Structured summary of trial design and methods, including items from the World Health Organization Trial Registration Data Set10	3
Protocol version	2	Version date and identifier	2, 12
Roles and responsibilities	3a	Names, affiliations, and roles of protocol contributors	1, 12
	3b	Name and contact information for the trial sponsor	1
	3c	Role of trial sponsor and funders in design, conduct, analysis, and reporting of trial; including any authority over these activities	12
	3d	Composition, roles, and responsibilities of the coordinating site, steering committee, endpoint adjudication committee, data management team, and other individuals or groups overseeing the trial, if applicable	11, 12
**Open science**			
Trial registration	4	Name of trial registry, identifying number (with URL), and date of registration. If not yet registered, name of intended registry	3
Protocol and statistical analysis plan	5	Where the trial protocol and statistical analysis plan can be accessed	3

Data sharing	6	Where and how the individual de-identified participant data (including data dictionary), statistical code, and any other materials will be accessible	11, 12
Funding and conflicts of interest	7a	Sources of funding and other support (eg, supply of drugs)	12
	7b	Financial and other conflicts of interest for principal investigators and steering committee members	12
Dissemination policy	8	Plans to communicate trial results to participants, healthcare professionals, the public, and other relevant groups (eg, reporting in trial registry, plain language summary, publication)	12
**Introduction**			
Background and rationale	9a	Scientific background and rationale, including summary of relevant studies (published and unpublished) examining benefits and harms for each intervention	2
	9b	Explanation for choice of comparator	2
Objectives	10	Specific objectives related to benefits and harms	2, 3
**Methods: Patient and public involvement, trial design**			
Patient and public involvement	11	Details of, or plans for, patient or public involvement in the design, conduct, and reporting of the trial	12
Trial design	12	Description of trial design including type of trial (eg, parallel group, crossover), allocation ratio, and framework (eg, superiority, equivalence, non-inferiority, exploratory)	3
**Methods: Participants, interventions, and outcomes**			
Trial setting	13	Settings (eg, community, hospital) and locations (eg, countries, sites) where the trial will be conducted	3
Eligibility criteria	14a	Eligibility criteria for participants	3, Supp 2
	14b	If applicable, eligibility criteria for sites and for individuals who will deliver the interventions (eg, surgeons, physiotherapists)	8, 9

Intervention and comparator	15a	Intervention and comparator with sufficient details to allow replication including how, when, and by whom they will be administered. If relevant, where additional materials describing the intervention and comparator (eg, intervention manual) can be accessed	8, Supp 5,6
	15b	Criteria for discontinuing or modifying allocated intervention/comparator for a trial participant (eg, drug dose change in response to harms, participant request, or improving/worsening disease)	9, 10
	15c	Strategies to improve adherence to intervention/comparator protocols, if applicable, and any procedures for monitoring adherence (eg, drug tablet return, sessions attended)	9
	15d	Concomitant care that is permitted or prohibited during the trial	9
Outcomes	16	Primary and secondary outcomes, including the specific measurement variable (eg, systolic blood pressure), analysis metric (eg, change from baseline, final value, time to event), method of aggregation (eg, median, proportion), and time point for each outcome	3-5, Tables 1, 2, 3, Supp 4
Harms	17	How harms are defined and will be assessed (eg, systematically, non-systematically)	11, Supp 10
Participant timeline	18	Time schedule of enrollment, interventions (including any run-ins and washouts), assessments, and visits for participants. A schematic diagram is highly recommended (see Figure)	6, 7 Figure 2, Table 3
Sample size	19	How sample size was determined, including all assumptions supporting the sample size calculation	10
Recruitment	20	Strategies for achieving adequate participant enrollment to reach target sample size	10, Supp 3
**Methods: Assignment of interventions**			
Randomization:			
Sequence generation	21a	Who will generate the random allocation sequence and the method used	8
	21b	Type of randomization (simple or restricted) and details of any factors for stratification. To reduce predictability of a random sequence, other details of any planned restriction (eg, blocking) should be provided in a separate document that is unavailable to those who enroll participants or assign interventions	8

Allocation concealment mechanism	22	Mechanism used to implement the random allocation sequence (eg, central computer/telephone; sequentially numbered, opaque, sealed containers), describing any steps to conceal the sequence until interventions are assigned	8
Implementation	23	Whether the personnel who will enroll and those who will assign participants to the interventions will have access to the random allocation sequence	8
Blinding	24a	Who will be blinded after assignment to interventions (eg, participants, care providers, outcome assessors, data analysts)	
	24b	If blinded, how blinding will be achieved and description of the similarity of interventions	8
	24c	If blinded, circumstances under which unblinding is permissible, and procedure for revealing a participant's allocated intervention during the trial	8
**Methods: Data collection, management, and analysis**			
Data collection methods	25a	Plans for assessment and collection of trial data, including any related processes to promote data quality (eg, duplicate measurements, training of assessors) and a description of trial instruments (eg, questionnaires, laboratory tests) along with their reliability and validity, if known. Reference to where data collection forms can be accessed, if not in the protocol	9, Supp 4
	25b	Plans to promote participant retention and complete follow-up, including list of any outcome data to be collected for participants who discontinue or deviate from intervention protocols	9
Data management	26	Plans for data entry, coding, security, and storage, including any related processes to promote data quality (eg, double data entry; range checks for data values). Reference to where details of data management procedures can be accessed, if not in the protocol	11
Statistical methods	27a	Statistical methods used to compare groups for primary and secondary outcomes, including harms	10, 11, Supp 8
	27b	Definition of who will be included in each analysis (eg, all randomized participants), and in which group	10, 11
	27c	How missing data will be handled in the analysis	10, 11

	27d	Methods for any additional analyses (eg, subgroup and sensitivity analyses)	10, 11
**Methods: Monitoring**			
Data monitoring committee	28a	Composition of data monitoring committee (DMC); summary of its role and reporting structure; statement of whether it is independent from the sponsor and funder; conflicts of interest and reference to where further details about its charter can be found, if not in the protocol. Alternatively, an explanation of why a DMC is not needed	11
	28b	Explanation of any interim analyses and stopping guidelines, including who will have access to these interim results and make the final decision to terminate the trial	11
Trial monitoring	29	Frequency and procedures for monitoring trial conduct. If there is no monitoring, give explanation	11
**Ethics**			
Research ethics approval	30	Plans for seeking research ethics committee/institutional review board approval	11, 12
Protocol amendments	31	Plans for communicating important protocol modifications to relevant parties	11, 12
Consent or assent	32a	Who will obtain informed consent or assent from potential trial participants or authorized proxies, and how	4
	32b	Additional consent provisions for collection and use of participant data and biological specimens in ancillary studies, if applicable	4
Confidentiality	33	How personal information about potential and enrolled participants will be collected, shared, and maintained in order to protect confidentiality before, during, and after the trial	11
Ancillary and post-trial care	34	Provisions, if any, for ancillary and post-trial care, and for compensation to those who suffer harm from trial participation	11

SUPPLEMENTAL MATERIAL 3

Box S3: Inclusion and exclusion criteria

Inclusion criteria

- Living in Australia or the United States
- Aged 18 years or older
- Diagnosed with IBS by a general practitioner, family physician, or gastroenterologist
- Currently fulfil Rome IV criteria for IBS
- Symptomatic at the time of recruitment (IBS-SSS ≥ 175)
- Access to a computer and internet
- Sufficient English language and computer skills to complete a text-based online treatment
- Willing to make dietary or behavioural changes in line with the allocated treatment programme
- Ability to provide informed consent

Exclusion criteria

- Presence or known history of other gastrointestinal disease (eg, coeliac disease, inflammatory bowel disease) or history of gastrointestinal cancer
- History of major gastrointestinal surgery (not appendectomy, cholecystectomy or haemorrhoidectomy)
- Individuals who report alarm symptoms (eg, blood in stool, recent unexplained/unintentional weight loss >5% body weight, a recent change in bowel habits if >50 years) will be excluded if appropriate medical investigations have not been conducted, unless written medical approval is provided from a general practitioner, family physician, or gastroenterologist.
- Diagnosis of major disease that could explain current gastrointestinal symptoms such as severe diabetic, cardiac, liver, neurological, neuropathy disease
- History or current diagnosis of psychotic disorder or bipolar disorder
- Current diagnosis of substance abuse disorder or major depressive disorder or active suicidal ideation
- History or current diagnosis of an eating disorder
- Current enteral/parenteral feeding or use of supplemental feeds
- Pregnant or lactating or planning to become pregnant during the 12-week intervention period
- BMI $\leq 18.5 kg/m^2$ or $\geq 34.9 kg/m^2$
- Unstable dose of antibiotics/medications that may affect the gastrointestinal transit (eg, anti-diarrhoeals and laxatives) in the 3 months preceding programme commencement.
- Unstable dose of probiotics, prebiotics, fibre supplements or digestive enzymes in the 3 months preceding programme commencement
- Commenced or change dose in psychotropic medication in the 3 months preceding programme commencement
- Currently undertaking behavioural therapy for IBS (including CBT), or have undertaken clinician-delivered behavioural therapy for the treatment of IBS
- Are currently undertaking or have undertaken a dietitian-prescribed and delivered restrictive diet for IBS (eg, FODMAP diet)

IBS-SSS, Irritable Bowel Syndrome-Symptom Severity Score; BMI, Body Mass Index

SUPPLEMENTAL MATERIAL 4

Box S4: Recruitment strategies

- **Online platforms:** Digital advertisements on university-affiliated and research websites (eg, University of Melbourne, Monash University FODMAP team), and relevant gastroenterology and general practice websites.
- **Flyers:** Hardcopy flyers distributed across university campuses, gastroenterology and medical clinics, and community locations (eg, noticeboards, libraries, shopping centres).
- **Social media:** Paid advertising and study-related posts across platforms (eg, Facebook, Instagram, X/Twitter, LinkedIn), including pages related to IBS, low FODMAP diets, and researcher networks.
- **Recruitment service:** A fee-for-service provider (Genesis Research Services) may be used to disseminate study advertisements via online and print channels.
- **Broadcast media:** Television and radio advertising.
- **Newspapers:** Advertisements in print and digital newspaper publications.

SUPPLEMENTAL MATERIAL 5

Table S5: Access and reminder process schedule

**Day 6 (Week 1)**	**Day 7 (Week 1)**	**Day 1 (Week 2)**	**Day 2 (Week 2)**	**Day 3 (Week 2)**	**Day 4 (Week 2)**	**Day 5 (Week 2)**	**Day 6 (Week 2)**	**Day 7 (Week 2)**
Intervention Module-week 1<br><br>Email: link to REDCap week 1 questionnaires	Intervention Module-week 1	Access to Intervention Module-week 2	Intervention Module-week 2<br><br>SMS: reminder for new week of programme and 2nd weekly food diary / stool sample	Intervention Module-week 2<br><br>Email: reminder for week 1 questionnaires	Intervention Module-week 2	Intervention Module-week 2<br><br>Email: reminder for week 1 questionnaires	Intervention Module-week 2<br><br>Email: link to week 2 questionnaires	Intervention Module-week 2<br><br>Email: link to REDCap week 2 questionnaires<br><br>Access to week 1 questionnaires removed

SUPPLEMENTAL MATERIAL 6

Table S6: Online FODMAP programme summary and schedule

Week (Module)	Diet Phase	Programme Content	Homework / Activities
1	N/A	Introduction to the programme	Brief summary of the FODMAP programme, instructions on how to navigate the CANVAS platform and how to interact with the dietitian.
1	N/A	Introduction to Irritable Bowel Syndrome and the FODMAP diet	4 short answer questions and 2 general comment questions.
2	Phase 1	Preparing for Phase 1 FODMAP diet: FODMAP restriction	5 activities (1 question), 4 short answer questions and 2 general comment questions<br>Activities:<br>1) Swap high FODMAP foods for low FODMAP foods.<br>2) Prepare a shopping list with low FODMAP foods.<br>3) Meal plan options with low FODMAP meals.<br>4) Modifying meals and recipes.<br>5) Symptom assessment question - rate overall gastrointestinal symptoms on a scale of 0 to 100.
3	Phase 1	Implementing Phase 1 of the FODMAP diet: FODMAP restriction	1 activity (question), 2 short answer questions and 2 general comment questions.<br>Activity:<br>1 multiple choice tick box question on how often the low FODMAP diet was followed over the past week.
4	Phase 1	Information and tips to help follow Phase 1 of the FODMAP diet	Same as above.
5	Phase 1	Information and tips to help follow Phase 1 of the FODMAP diet	Same as above.
6	Phase 1	Additional recipes and assessing symptom response to Phase 1 of the FODMAP diet	3 activities (questions), 1 short answer question and 1 general comment question.<br>Activities:<br>1) 1 multiple choice tick box question on how often the low FODMAP diet was followed over the past week.<br>2) Symptom assessment question - rate overall gastrointestinal symptoms on a scale of 0 to 100.

			3) Symptom assessment question - rate how much symptoms changed on a scale of -10 to 10.
7	Phase 2	Phase 2 of the FODMAP diet: FODMAP reintroduction	1 activity, 4 short answer questions and 2 general comment questions<br>Activity:<br>Challenge plan - plan what FODMAP type/s, food and the amounts to challenge during Phase 2 of the FODMAP reintroduction phase.
8	Phase 2	Review of key steps to help follow Phase 2 of the FODMAP diet	4 short answer questions and 2 general comment questions.
9	Phase 2	Review of key steps to help follow Phase 2 of the FODMAP diet	4 short answer questions and 2 general comment questions.
10	Phase 2 / Phase 3	Phase 3 of the FODMAP diet: FODMAP personalisation<br>(May still be completing Phase 2 FODMAP reintroduction)	1 activity (2 questions) and 3 short answer questions and 2 general comment questions.<br>Activity:<br>2 questions related to FODMAP personalisation<br>1) List one or more food challenges<br>2) List 1-2 situations where it feels comfortable reintroducing the foods.
11	Phase 2 / Phase 3	Review of key steps to help follow Phase 2 or Phase 3 of the FODMAP diet<br>(May still be completing Phase 2 FODMAP reintroduction)	3 short answer questions and 2 general comment questions.
12	Phase 2 / Phase 3	Completing the 12-week online treatment programme<br>(May still be completing Phase 2 FODMAP reintroduction)	6 short answer questions and 1 general comment question.

SUPPLEMENTAL MATERIAL 7

Table S7: Online E-CBT programme summary and schedule

Week (Module)	Programme Steps	Programme Content	Homework / Activities
1	N/A	Introduction to the programme	Brief summary of the E-CBT programme, instructions on how to navigate the CANVAS platform and how to interact with the psychologist.
1	Step 1	The Treatment Programme, Monitoring of the Body and Mindfulness	3 activities, 8 short answer questions and 1 general comment question.<br>Activities:<br>1) Practice 'Self-Observation in 15 Minutes'<br>2) Practice 'Self-Observation in Everyday Life'<br>3) Fill in 'My IBS Behaviors Worksheet'
2	Step 2	When the Gut Becomes a Threat	5 activities, 8 short answer questions and 1 general comment question.<br>Activities:<br>1) Practice 'Self-Observation in 15 Minutes'<br>2) Practice 'Self-Observation in Everyday Life'<br>3) Practice 'Self-Observation When You Have Symptoms'<br>4) Update 'My IBS Behaviors Worksheet'<br>5) Read 'Questions and Answers about Self-Observation'
3	Step 3	Thoughts, Behaviours and Focusing on Life Values	4 activities, 11 short answer questions and 1 general comment question.<br>Activities:<br>1) Practice 'Self-Observation in 15 Minutes'<br>2) Practice 'Self-Observation in Everyday Life'<br>3) Practice 'Self-Observation When You Have Symptoms'<br>4) Update 'My IBS Behaviors Worksheet'
4	Step 4	Exposure	6 activities, 10 short answer questions (2 questions to report on the activity - plan and execute 2 exposure exercises) and 1 general comment question.<br>Activities:<br>1) Practice 'Postponing Toilet Visits' and/or 'Scheduling Toilet Visits' and fill in worksheet/s<br>2) Practice 'Self-Observation in 15 Minutes'<br>3) Practice 'Self-Observation in Everyday Life'<br>4) Practice 'Self-Observation When You Have Symptoms'<br>5) Plan and execute two exposures from the 'My IBS Behaviors Worksheet'

			6) Read 'Treatment Examples'
5	Step 5	Exposure in Practice – Reports 1-12<br>Includes: (1) exercises that provoke symptoms, (2) abolishment of behaviours that serve to control symptoms, and/or (3) exposure to situations where symptoms were unwanted.	5-6 activities, plan exposure exercises (8 short answer questions (3 questions to plan 3 exposure challenges) and 1 general question).<br>Activities:<br>1) Practice 'Postponing Toilet Visits' and/or 'Scheduling Toilet Visits' and fill in worksheet/s<br>2) Practice 'Self-Observation in 15 Minutes'<br>3) Practice 'Self-Observation in Everyday Life'<br>4) Practice 'Self-Observation When You Have Symptoms'<br>5) Start exposure challenges<br>6) Start Exposure Report 1 (optional)
6	Step 5		5 activities and minimum of 1 exposure report submitted per week (3 questions on self-observation and toilet visit activities, 23 questions to report on how the exposure challenges went, 3 questions to plan for next exposure challenges and 1 general question).<br>Activities:<br>1) Complete planned exposure challenges<br>2) Practice 'Postponing Toilet Visits' and/or 'Scheduling Toilet Visits' and fill in worksheet/s<br>3) Practice 'Self-Observation in 15 Minutes'<br>4) Practice 'Self-Observation in Everyday Life'<br>5) Practice 'Self-Observation When You Have Symptoms'
7	Step 5		
8	Step 5		
9	Step 5		
10	Step 5		
11	Step 5		
12	Step 6	Completing the treatment	7 short answer questions and 1 general comment question.

SUPPLEMENTAL MATERIAL 8

Assessment of participant adherence to online programmes

FODMAP diet programme

Phase 1: Restriction (weeks 3–6)

Adherence to the restriction phase will be assessed using weekly homework responses to a single self-report item: *"How often did you follow the low FODMAP diet over the past week (ie, only included low FODMAP foods)?"* Response options are provided on a 4-point Likert scale: <25%, 25–50%, 51–75%, and 76–100% of the time[27].

Participants will be classified as adherent if they report following the diet ≥50% of the time (ie, "frequently" or "always") on at least 2 of the 4 weekly assessments[28]. Objective adherence will also be assessed at week 6 using 3-day food diary data. Participants will be classified as adherent if total FODMAP intake is ≤12 g/day or reduced by ≥30% from baseline[29].

Phase 2: Reintroduction (weeks 7–12)

Adherence to the reintroduction phase will be assessed using weekly homework responses documenting food challenges undertaken. Participants are asked to report the FODMAP type, food tested, and doses consumed for each food challenge[29]. Participants will be classified as adherent if they complete at least one food challenge per week in accordance with the programme protocol, during weeks 7–12 or until transition to phase 3.

Phase 3: Personalisation (follow-up)

Adherence to the personalisation phase will be assessed at 3- and 6-month follow-up using a single self-report item reflecting current dietary practice[29-31]. Response options include:
(i) continued strict low FODMAP diet;
(ii) reintroduced high-FODMAP foods to tolerance (personalisation phase);
(iii) partial adherence (approximately 50% restriction);
(iv) return to habitual diet.

E-CBT programme

Adherence to the E-CBT programme will be assessed using self-reported completion of core treatment components recorded via weekly homework responses. These include self-

observation exercises and behavioural exposure activities (toilet behaviour and IBS-related behaviours).

Weeks 1–4 (self-observation phase)

Participants report the number of completed self-observation exercises across three different observation formats (Self-Observation in 15 Minutes; Self-Observation in Everyday Life; and Self-Observation When You Have Symptoms). Participants will be classified as adherent if they report completing at least one self-observation exercise per week.

Weeks 5–12 (exposure phase)

Adherence will be assessed using weekly "exposure reports" documenting completion of toilet behaviour and IBS-related exposure exercises. Participants will be classified as adherent if they report completing at least one exposure exercise per week for at least 5 of the 8 weeks (weeks 5–12).

SUPPLEMENTAL MATERIAL 9

Mediation analyses confirmatory criteria

Between-group mediation (parallel process latent growth curve modelling)

The hypothesis of differential mechanisms across the two treatments will be considered confirmed if (a) each of the three mediators (FODMAP intake, gastrointestinal-specific anxiety, and avoidance behaviour) shows a statistically significant indirect effect on the outcome, and (b) the indirect effect via the FODMAP-specific mediator (FODMAP intake) is opposite in sign to the indirect effects via the E-CBT-specific mediators (gastrointestinal-specific anxiety and avoidance behaviour).

Within-group time-lagged associations (random intercentp cross-lagged panel models; RI-CLPM)

For these analyses, the hypothesis of differential mechanisms across the two treatments will be considered confirmed if (a) in the FODMAP group: FODMAP intake at week T predicts gastrointestinal symptoms at week T+1, whereas gastrointestinal-specific anxiety and avoidance behaviour at week T do not predict gastrointestinal symptoms at week T+1; and (b) in the E-CBT group: gastrointestinal-specific anxiety and avoidance behaviour at week T predict gastrointestinal symptoms at week T+1, whereas FODMAP intake at week T does not predict gastrointestinal symptoms at week T+1.

SUPPLEMENTAL MATERIAL 10

Microbiome data collection and analyses

Stool samples will be collected according to standardised instructions using OMNIgene GUT collection tubes (OM-200, Go Lab, Australia) and stored at -80°C until analysis. Stool DNA will be isolated using QIAamp Fast DNA Stool Mini Kit or AllPrep DNA/RNA Mini Kit (Qiagen kits; Qiagen Pty Ltd, Victoria, Australia) according to manufacturer instructions.

Gut microbiome composition and function will be assessed using full-length 16S rRNA gene amplicon sequencing and shotgun metagenomic sequencing. Bioinformatic analysis will be conducted using R and RStudio, following the planned approach below.

For 16S rRNA sequencing, amplicon sequence variants will be generated to characterise microbial composition. Alpha diversity (Observed, Chao1, ACE, Shannon, Simpson, InvSimpson, Fisher) and beta diversity (weighted and unweighted UniFrac, Bray-Curtis dissimilarity, visualised via principal coordinate analysis) will be calculated. Permutational multivariate analysis of variance will assess between-group differences in community composition, and DESeq2 will identify differentially abundant genera.

For shotgun metagenomic sequencing, libraries will be prepared using the Illumina Nextera XT DNA Library Preparation Kit and sequenced on an Illumina NovaSeq 6000 platform. Taxonomic and functional annotation will be performed using tools such as Kraken2, MetaPhlAn, and HUMAnN, referenced against the NCBI RefSeq database, to assess microbial composition, functional pathways, and species-level identification. Comparative analyses will identify microbial signatures associated with IBS and treatment response (FODMAP diet versus E-CBT). Bioinformatic pipieline versions will be reported in accordance with STORMS (Strengthening The Organising and Reporting of Microbiome Studies) reporting guidelines[32].

SUPPLEMENTAL MATERIAL 11

Dietary intake data collection and analyses

Dietary intake will be assessed every two weeks using 3-day food diaries (including one weekend day) and recorded using paper-based methods or a mobile application (Easy Diet Diary, Xyris Software Pty Ltd, Australia). Participants will be provided with standardised instructions on how to complete food diaries, including guidance on reporting food types, portion sizes, preparation methods, and mixed dishes and takeaway foods. Missing or incorrect information will be requested from participants within two days of diary submission.

Food diary data will be reviewed by trained members of the research team (dietitians or trained dietetic/food science students) to optimise completeness and accuracy. Two reminders will be sent for this information after which time the information will be deemed missing or incomplete. Standardised procedures will be used for data entry, cleaning, and handling of incomplete or ambiguous entries, including the application of predefined rules to estimate missing quantities where necessary.

Dietary intake will be analysed using the Monash University FODMAP Calculator (Monash University; Melbourne, Australia) and FoodWorks software (Xyris Software Pty, Ltd, Brisbane, Australia). Total FODMAP intake (g/day) will be calculated as the sum of individual FODMAP components, including excess fructose, lactose, fructans, galacto-oligosaccharides (GOS), and polyols (mannitol and sorbitol), and averaged across recording days. Energy, macronutrient, and fibre intake will also be assessed.

SUPPLEMENTAL MATERIAL 12

Table S12: Safety monitoring and reporting definitions

**Adverse Event (AE)**	Any untoward medical occurrence in a participant enrolled into this study regardless of its causal relationship to study treatment.
**Serious Adverse Event (SAE)**	Any adverse event that: results in death; is life threatening; requires >24 hours hospitalisation or prolongation of existing hospitalisation; results in persistent or significant disability or incapacity; or is a congenital anomaly or birth defect.
**Relationship to trial treatment of the AE**	**Unrelated**: There is no association between the trial intervention and the reported event.<br>**Possible**: The event could have caused or contributed to the AE.<br>**Probable**: The association of the event with the trial intervention seems likely.<br>**Definite**: The AE is a consequence of administration of the trial intervention. AEs in the category cannot be explained by concurrent illness, progression of disease state or concurrent medication reaction.
**Severity of the AE**	**Mild**: Events that require minimal or no treatment and do not interfere with the participant's daily activities.<br>**Moderate**: Events that cause sufficient discomfort to interfere with daily activity and/or require a simple dose of medication.<br>**Severe**: Events that prevent usual daily activity or require complex treatment.

REFERENCES

1. Palsson OS, Whitehead WE, van Tilburg MA, et al. Rome IV Diagnostic Questionnaires and Tables for Investigators and Clinicians. *Gastroenterology* 2016 doi: 10.1053/j.gastro.2016.02.014
2. Lacy BE, Patel NK. Rome Criteria and a Diagnostic Approach to Irritable Bowel Syndrome. *J Clin Med* 2017;6(11) doi: 10.3390/jcm6110099
3. Francis CY, Morris J, Whorwell PJ. The irritable bowel severity scoring system: a simple method of monitoring irritable bowel syndrome and its progress. *Aliment Pharmacol Ther* 1997;11(2):395-402. doi: 10.1046/j.1365-2036.1997.142318000.x
4. Fairburn CG, Beglin SJ. Assessment of eating disorders: interview or self-report questionnaire? *Int J Eat Disord* 1994;16(4):363-70.
5. Machado PPP, Grilo CM, Rodrigues TF, et al. Eating Disorder Examination - Questionnaire short forms: A comparison. *Int J Eat Disord* 2020;53(6):937-44. doi: 10.1002/eat.23275
6. Kroenke K, Spitzer RL, Williams JB. The PHQ-9: validity of a brief depression severity measure. *J Gen Intern Med* 2001;16(9):606-13. doi: 10.1046/j.1525-1497.2001.016009606.x
7. Blake MR, Raker JM, Whelan K. Validity and reliability of the Bristol Stool Form Scale in healthy adults and patients with diarrhoea-predominant irritable bowel syndrome. *Aliment Pharmacol Ther* 2016;44(7):693-703. doi: 10.1111/apt.13746
8. Wiklund IK, Fullerton S, Hawkey CJ, et al. An irritable bowel syndrome-specific symptom questionnaire: development and validation. *Scand J Gastroenterol* 2003;38(9):947-54. doi: 10.1080/00365520310004209
9. Ljótsson B, Jones M, Talley NJ, et al. Discriminant and convergent validity of the GSRS-IBS symptom severity measure for irritable bowel syndrome: A population study. *United European Gastroenterol J* 2020;8(3):284-92. doi: 10.1177/2050640619900577
10. Wallén H, Ljótsson B, Svanborg C, et al. Exposure based cognitive behavioral group therapy for IBS at a gastroenterological clinic - a clinical effectiveness study. *Scand J Gastroenterol* 2022;57(8):904-11. doi: 10.1080/00365521.2022.2047220
11. Labus JS, Mayer EA, Chang L, et al. The central role of gastrointestinal-specific anxiety in irritable bowel syndrome: further validation of the visceral sensitivity index. *Psychosom Med* 2007;69(1):89-98. doi: 10.1097/PSY.0b013e31802e2f24

12. Labus JS, Bolus R, Chang L, et al. The Visceral Sensitivity Index: development and validation of a gastrointestinal symptom-specific anxiety scale. *Aliment Pharmacol Ther* 2004;20(1):89-97. doi: 10.1111/j.1365-2036.2004.02007.x
13. Reme SE, Darnley S, Kennedy T, et al. The development of the irritable bowel syndrome-behavioral responses questionnaire. *J Psychosom Res* 2010;69(3):319-25. doi: 10.1016/j.jpsychores.2010.01.025
14. Burton Murray H, Dreier MJ, Zickgraf HF, et al. Validation of the nine item ARFID screen (NIAS) subscales for distinguishing ARFID presentations and screening for ARFID. *Int J Eat Disord* 2021;54(10):1782-92. doi: 10.1002/eat.23520
15. Zickgraf HF, Ellis JM. Initial validation of the Nine Item Avoidant/Restrictive Food Intake disorder screen (NIAS): A measure of three restrictive eating patterns. *Appetite* 2018;123:32-42. doi: 10.1016/j.appet.2017.11.111 [published Online First: 20171205]
16. Zickgraf HF, Loftus P, Gibbons B, et al. "If I could survive without eating, it would be a huge relief": Development and initial validation of the Fear of Food Questionnaire. *Appetite* 2022;169:105808. doi: 10.1016/j.appet.2021.105808
17. Patrick DL, Drossman DA, Frederick IO, et al. Quality of life in persons with irritable bowel syndrome: development and validation of a new measure. *Dig Dis Sci* 1998;43(2):400-11. doi: 10.1023/a:1018831127942
18. Spitzer RL, Kroenke K, Williams JB, et al. A brief measure for assessing generalized anxiety disorder: the GAD-7. *Arch Intern Med* 2006;166(10):1092-7. doi: 10.1001/archinte.166.10.1092
19. Kroenke K, Spitzer RL, Williams JB. The PHQ-15: validity of a new measure for evaluating the severity of somatic symptoms. *Psychosom Med* 2002;64(2):258-66. doi: 10.1097/00006842-200203000-00008
20. Polster AV, Palsson OS, Törnblom H, et al. Subgroups of IBS patients are characterized by specific, reproducible profiles of GI and non-GI symptoms and report differences in healthcare utilization: A population-based study. *Neurogastroenterol Motil* 2019;31(1):e13483. doi: 10.1111/nmo.13483
21. Devilly GJ, Borkovec TD. Psychometric properties of the credibility/expectancy questionnaire. *J Behav Ther Exp Psychiatry* 2000;31(2):73-86. doi: 10.1016/s0005-7916(00)00012-4

22. Gómez Penedo JM, Berger T, Grosse Holtforth M, et al. The Working Alliance Inventory for guided Internet interventions (WAI-I). *J Clin Psychol* 2020;76(6):973-86. doi: 10.1002/jclp.22823
23. Uebelacker LA, Weinstock LM, Battle CL, et al. Treatment credibility, expectancy, and preference: Prediction of treatment engagement and outcome in a randomized clinical trial of hatha yoga vs. health education as adjunct treatments for depression. *J Affect Disord* 2018;238:111-17. doi: 10.1016/j.jad.2018.05.009
24. Herrero R, Vara MD, Miragall M, et al. Working Alliance Inventory for Online Interventions-Short Form (WAI-TECH-SF): The Role of the Therapeutic Alliance between Patient and Online Program in Therapeutic Outcomes. *Int J Environ Res Public Health* 2020;17(17) doi: 10.3390/ijerph17176169
25. Attkisson CC, Zwick R. The client satisfaction questionnaire. Psychometric properties and correlations with service utilization and psychotherapy outcome. *Eval Program Plann* 1982;5(3):233-7. doi: 10.1016/0149-7189(82)90074-x
26. Larsen DL, Attkisson CC, Hargreaves WA, et al. Assessment of client/patient satisfaction: development of a general scale. *Eval Program Plann* 1979;2(3):197-207. doi: 10.1016/0149-7189(79)90094-6
27. Shepherd SJ, Gibson PR. Fructose malabsorption and symptoms of irritable bowel syndrome: guidelines for effective dietary management. *J Am Diet Assoc* 2006;106(10):1631-9. doi: 10.1016/j.jada.2006.07.010
28. Staudacher HM, Lomer MCE, Farquharson FM, et al. A Diet Low in FODMAPs Reduces Symptoms in Patients With Irritable Bowel Syndrome and A Probiotic Restores Bifidobacterium Species: A Randomized Controlled Trial. *Gastroenterology* 2017;153(4):936-47. doi: 10.1053/j.gastro.2017.06.010
29. Tuck CJ, Reed DE, Muir JG, et al. Implementation of the low FODMAP diet in functional gastrointestinal symptoms: A real-world experience. *Neurogastroenterol Motil* 2020;32(1):e13730. doi: 10.1111/nmo.13730
30. Rej A, Shaw CC, Buckle RL, et al. The low FODMAP diet for IBS; A multicentre UK study assessing long term follow up. *Dig Liver Dis* 2021;53(11):1404-11. doi: 10.1016/j.dld.2021.05.004
31. Weynants A, Goossens L, Genetello M, et al. The long-term effect and adherence of a low fermentable oligosaccharides disaccharides monosaccharides and polyols (FODMAP)

diet in patients with irritable bowel syndrome. *J Hum Nutr Diet* 2020;33(2):159-69. doi: 10.1111/jhn.12706

32. Mirzayi C, Renson A, Zohra F, et al. Reporting guidelines for human microbiome research: the STORMS checklist. *Nat Med* 2021; 27(11):1885-92. doi:10.1038/s41591-021-01552-x.
