# Supplementary material for: Exclude or expose in irritable bowel syndrome study: what works for whom and how? Protocol for a randomised controlled trial of internet-delivered FODMAP diet versus exposure-based cognitive behavioural therapy
Source: BMJ Open. 2026 Nov 11;16(11):e126664. doi: 10.1136/bmjopen-2026-126664 (PMC13756487; doi:10.1136/bmjopen-2026-126664)
Supplement: Supplementary file 2 [file bmjopen-2026-126664.reviewer_comments.pdf]

PEER REVIEW HISTORY

BMJ Open publishes all reviews undertaken for accepted manuscripts. Reviewers are asked to complete a checklist review form and are provided with free text boxes to elaborate on their assessment. These free text comments are reproduced below.

ARTICLE DETAILS

Title (Provisional)

Exclude or Expose in Irritable Bowel Syndrome Study: what works for whom and how? Protocol for a randomised controlled trial of internet-delivered FODMAP diet versus exposure-based cognitive behavioural therapy

Authors

Anderson, Jacqueline Louise; Ljótsson, Brjánn; Burton-Murray, Helen; West, Madeline L; Yao, Chu; Basnayake, Chamara; Van Oudenhove, Lukas; Biesiekierski, Jessica

---

VERSION 1 - REVIEW

**Reviewer**	**1**
**Name**	**Jackson, Tanya**
**Affiliation**	**University of Alberta, Medicine**
**Date**	**02-Oct-2026**
**COI**	**none**

Thank you for the opportunity to read this. This is a well written and interesting protocol and I have no suggestions for improvement on much of the content. A few questions to consider:

1. What is the difference between 12 weeks and 3 months, for followup?

2. In the FODMAP intervention, patients will receive feedback from a therapist even though this is a dietary focused intervention?

3. There is also a dietitian available to consult - will this lead to more participant support in the FODMAP condition than in the other treatment condition?

4. The explanation on how you will statistically analyze the results appears carefully planned and detailed. The explanation of the qualitative analysis suffers by comparison.

5. Line 241 - should this be constraints?

6. In line 270, you say only that any new therapy relevant to IBS is recorded as a protocol deviation, but in the protocol deviation section line 365, you state new therapies that interfere with the allocated intervention may result in discontinuation, while others that do

not affect participation are reviewed at team meetings. Perhaps clarify is it recorded? recorded and reviewed? other action taken? and under which circumstances? for consistency.

7. Line 496, you state adverse events will be escalated to participants' usual clinician as required. Did you or do you plan to collect consent from participants to contact their regular clinicians? If not, should you edit this to state you will encourage participants to escalate this to their usual clinicians?

VERSION 1 - AUTHOR RESPONSE

Reviewer 1 (Dr Tanya Jackson) comments

1. What is the difference between 12 weeks and 3 months, for follow-up?

Author response: Thank you for highlighting this ambiguity. "12 weeks" is the end-of-intervention (post-treatment) assessment, measured from program commencement. The 3- and 6-month follow-ups are measured from the *end* of the intervention

We have clarified this throughout:

- *Abstract (Methods and analysis), line 50: "3- and-6-month follow-up" changed to "3- and 6-month post-intervention follow-up".*
- *Study design and setting, line 209: "Follow-up assessments will be conducted at 3 and 6 months after completion of the 12-week intervention."*
- *Table 1 and Table 2 footnotes: added a note that follow-up assessments are conducted 3 and 6 months after completion of the 12-week intervention.*

2. In the FODMAP intervention, patients will receive feedback from a therapist even though this is a dietary focused intervention?

Author response: Yes. We use "therapist" as an umbrella term for the treating clinician in each arm: an Accredited Practising Dietitian in the FODMAP arm and a Clinical Psychologist in the E-CBT arm. In the FODMAP arm, the dietitian reviews weekly homework (for example, restriction adherence and the results of reintroduction challenges) and provides dietary feedback.

We have made this explicit:

- *Interventions – Overview, line 296:* added "Throughout, 'therapist' refers to the treating clinician in each arm: an Accredited Practising Dietitian (FODMAP diet) or a Clinical Psychologist (E-CBT)."
- *Abstract, line 42:* "each with therapist support" changed to "each with therapist support (dietitian or psychologist, respectively)".

3. There is also a dietitian available to consult - will this lead to more participant support in the FODMAP condition than in the other treatment condition?

Author response: Thank you for raising this. The dietitian is the FODMAP-arm therapist, not an additional source of support; there is no separate dietitian consultation. Each arm receives asynchronous support from one clinician of the relevant discipline, matched for contact time (approximately 15 minutes per participant per week), mode, platform and message-bank guidance. Therapists log weekly contact time per participant, and the average is reviewed regularly to confirm it remains comparable between arms. The definition of "therapist" added in response to Comment 2 (line 297) clarifies this in the manuscript.

4. The explanation on how you will statistically analyze the results appears carefully planned and detailed. The explanation of the qualitative analysis suffers by comparison.

Author response: We agree and have expanded the description of the qualitative component under a new subheading, Qualitative analysis (lines 518-529), specifying the timing, scope, data handling, analytic approach and reporting standard. The previous brief paragraph in Methods of analysis has been removed. As the interview guide and detailed procedures are still in development, we have stated this explicitly and noted that they will be finalised before data collection and reported in full in the qualitative results paper.

The new subsection reads:

"A nested qualitative study will explore participants' experiences of the interventions. All participants will be invited to a semi-structured interview at 6-month follow-up conducted by a trained researcher. Interviews will broadly explore intervention experience and acceptability, perceived mechanisms of change, and use of intervention strategies during follow-up. The interview guide and detailed procedures (including sampling) are under development, will be informed by emerging trial processes and co-investigator input, and will be finalised and submitted for ethics approval before qualitative data collection commences.

Interviews will be audio-recorded, transcribed verbatim, de-identified and analysed using reflexive thematic analysis supported by NVivo software. Findings will be reported in line with the Consolidated Criteria for Reporting Qualitative Research (COREQ) and, where informative, integrated with quantitative outcomes to contextualise treatment response."

5. Line 241 - should this be "constraints"?

Author response: Thank you; this has been corrected. We have also corrected other typographical errors:

- In *Baseline assessments*, changed "resource constrains" to "resource constraints"
- "Trial oversite" to "Trial oversight"
- "identificaton" to "identification"

6. In line 270, you say only that any new therapy relevant to IBS is recorded as a protocol deviation, but in the protocol deviation section line 365, you state new therapies that interfere with the allocated intervention may result in discontinuation, while others that do not affect participation are reviewed at team meetings. Perhaps clarify is it recorded? recorded and reviewed? other action taken? and under which circumstances? for consistency.

Author response: Thank you. We have harmonised the two sections and clarified that protocol deviations apply only to the 12-week intervention period; new treatments commenced during follow-up are recorded but are not considered protocol deviations.

- *Trial procedures and follow-up, lines 273-274:* changed to "…new IBS-related therapies are recorded via the protocol compliance questionnaire but are not considered protocol deviations (see Protocol deviations)."
- *Protocol deviations, lines 378-385:* revised to read: *"Participants are asked to maintain baseline treatments (medications, supplements, therapies) throughout the 12-week intervention where possible. New treatments are recorded weekly via the protocol compliance questionnaire and reviewed by the study team. During the intervention, commencing a new (non-study) dietitian-led IBS restriction diet or clinician-delivered IBS behavioural therapy is considered a protocol deviation that may interfere with the allocated intervention and may result in discontinuation, as determined by the Trial Management Group; these participants will be encouraged to continue study*

assessments. Other new treatments that do not affect intervention participation are recorded as minor protocol deviations and reviewed at team meetings. New treatments commenced during the 3- and 6-month follow-up are recorded but are not considered protocol deviations."

7. Line 496, you state adverse events will be escalated to participants' usual clinician as required. Did you or do you plan to collect consent from participants to contact their regular clinicians? If not, should you edit this to state you will encourage participants to escalate this to their usual clinicians?

Author response: Thank you. To protect participant confidentiality, the study team does not contact participants' clinicians directly; participants are instead advised to seek review from their usual clinician. We have revised the text accordingly (Safety monitoring and reporting, line 559): "Intervention-related adverse events will be managed as described above, and participants will be advised to seek review from their usual clinician, or emergency care where appropriate."
